# Developing Cardiothoracic Surgical Critical Care Intensivists: A Case for Distinct Training

**DOI:** 10.3390/medicina58121865

**Published:** 2022-12-17

**Authors:** Rafal Kopanczyk, Micah T. Long, Sree V. Satyapriya, Amar M. Bhatt, Michael Lyaker

**Affiliations:** 1 Department of Anesthesiology, The Ohio State University Wexner Medical Center, Columbus, OH 43210, USA; 2 Department of Anesthesiology, University of Wisconsin Hospitals & Clinics, Madison, WI 53792, USA

**Keywords:** education, cardiothoracic surgery, intensive care medicine, anesthesiology

## Abstract

Cardiothoracic surgical critical care medicine is practiced by a diverse group of physicians including surgeons, anesthesiologists, pulmonologists, and cardiologists. With a wide array of specialties involved, the training of cardiothoracic surgical intensivists lacks standardization, creating significant variation in practice. Additionally, it results in siloed physicians who are less likely to collaborate and advocate for the cardiothoracic surgical critical care subspeciality. Moreover, the current model creates credentialing dilemmas, as experienced by some cardiothoracic surgeons. Through the lens of critical care anesthesiologists, this article addresses the shortcomings of the contemporary cardiothoracic surgical intensivist training standards. First, we describe the present state of practice, summarize past initiatives concerning specific training, outline why standardized education is needed, provide goals of such training standardization, and offer a list of desirable competencies that a trainee should develop to become a successful cardiothoracic surgical intensivist.

## 1. Introduction

Cardiothoracic surgical critical care medicine (CT-CCM) remains unrecognized as a specific subspeciality of critical care medicine (CCM) [1]. Nonetheless, CT-CCM is currently practiced by a diverse group, including anesthesiologists, cardiothoracic surgeons, internists, cardiologists, and emergency department physicians [2]. As a result, there is a marked variation in CT-CCM training between specialties and fellowship programs, with an overall lack of standardization [1,2,3,4]. For example, anesthesiology critical care fellows typically spend 2–6 months in cardiothoracic intensive care units (CT-ICUs) during training [5]. In contrast, internal medicine critical care fellows rotate through CT-ICUs in the second year of training on an elective and optional basis. Cardiothoracic surgery (CTS) fellows, on the other hand, round and follow their ICU patients throughout the entirety of CTS training, but specific critical care education varies significantly and, accordingly, critical care knowledge may be inadequate. Further, there is no official requirement for ICU rotations or rotations under CCM trained faculty in the traditional training pathway. Instead, cardiothoracic fellows who have the desire to practice independently in CT-CCM typically enter a surgical critical care fellowship and focus their experience on CT-CCM [6,7,8]. Consequently, CT-CCM training is subject to institutional culture, in-unit expertise, and specialty-related biases.

The lack of standardization and official recognition of CT-CCM has significant ramifications. First, competency in CT-CCM skills varies between graduates of different programs, creating inconsistent standards and quality of CT-CCM care. Second, intensivists siloed by particular specialties are less likely to collaborate and advocate for CT-CCM as a whole. Compartmentalized critical care experiences–either within only CT-CCM (e.g., for cardiothoracic fellows), or only medical ICU experiences (e.g., some medical fellows)-limits growth and stifles the scientific inquiry needed to advance CT-CCM. Lastly, credentialing difficulties can result where, for example, cardiothoracic surgeons without an official CCM fellowship are unable to gain ICU privileges [6].

As a result, overcoming these limitations in CT-CCM intensivists training requires strong leadership. While cardiothoracic surgeons often claim ownership of the CT-CCM realm because of an appropriate sense of responsibility for patients, on whom they operated, and perceived expertise in the field, the delineation of training standards required to achieve competency in CT-CCM or standardizing dual training in critical care and CT surgery has not occurred [2,3,4,6,9]. On the other hand, critical care anesthesiology has been expanding in the U.S. with a typical emphasis on practice in cardiothoracic critical care [10]. Notably, the coronavirus disease 2019 (COVID-19) pandemic further brought anesthesiologists to the forefront of critical care due to successful leadership in initiatives, such as CAESAR-ICU, anesthesia machine repurposing, and volunteer staffing of COVID-19 intensive care units [11,12]. Moreover, critical care anesthesiologists expanded their specialty’s footprint in cardiothoracic surgical critical care units (CT-ICUs) by providing very specific care for the sickest COVID-19 population, including care of patients supported with extra-corporeal membrane oxygenation (ECMO) and lung transplantation recipients [13]. Therefore, the specialty of anesthesiology has a unique and historical opportunity to continue the innovative approach for critical care exemplified during the pandemic.

Anesthesiologists are well-positioned to lead cardiothoracic CT-CCM initiatives. General, cardiothoracic, and critical care anesthesiology training provide physicians with skills and the bandwidth distinctively suited for CT-ICU care [1,14,15]. Moreover, anesthesiologists presently make up a large fraction of CT-CCM intensivists and are particularly adept at multidisciplinary collaboration, which is crucial to the outcomes in the CT-ICU [10]. Through the lens of critical care anesthesiology, this article proposes the reimaging of CT-CCM training. We describe the present state of CT-CCM practice, summarize past initiatives concerning specific CT-CCM training, outline why standardized CT-CCM education is needed, provide goals for such training standardization, and offer a list of desirable competencies that a trainee should develop to become a successful CT-CCM intensivist.

## 2. Current Practice in CT-ICUs in the United States

CT-ICUs are hospital wards where multidisciplinary teams provide highly specialized medical care for critically ill patients [16]. These units deal with unique pathophysiologies, distinct surgical sequalae, and novel mechanical therapies [17]. Most CT-ICU patients are recovering from surgical procedures involving the thoracic cavity, including coronary bypass grafting, valve replacement, lung transplantation, pneumonectomy, and complicated transcutaneous aortic valve replacement, amongst others [1,8,17]. Moreover, other surgical services also recover their patients in CT-ICUs, as is often the case with vascular surgery patients. However, a subset of CT-ICU patients are non-surgical and treated in the unit for severe respiratory or cardiac failure, necessitating extra-corporeal life support (ECLS) or other circulatory support devices [18].

The professionals managing these patients have changed over the years [17]. Initially, the CT-ICU model of care involved attending surgeons rounding before or after scheduled operations with surgical trainees, consultants, trained nursing staff, and other technicians enacting dictated care plans. Recently, the open and semi-open ICU models have become more common with physician intensivists assuming primary roles in perioperative care [16]. This change was initially driven by trainee workhour limitations but is now becoming the standard-of-care due to improved patient outcomes [17,19,20,21,22,23,24,25,26].

## 3. Past Initiatives concerning CT-CCM Education and Training

The distinct pathophysiologies, complexity of care, and technological innovations make specific CT-CCM training essential, yet the ideal credentials and expertise required to practice in the CT-ICU remain undefined [16,17]. Indeed, an adequate duration of directed training and education guided by expert mentors in the field is required [15,27,28,29]. The need for exclusive preparation was acknowledged from the early days of open-heart surgery, where considerable time was dedicated to talent acquisition and on-the-job nurse training at Stanford and Mayo Clinic in the 1950’s [30,31]. Additionally, it was recognized that postoperative physician presence was crucial, with cardiac surgeon Dr. Dwight McGoon, likely the first cardiothoracic intensivist, tending to patients at the bedside continuously for up to 12 h at Mayo Hospital [30]. However, founding CT-CCM physicians did not translate their experience and learning pathway into the development of an exclusive CT-CCM subspeciality or associated competency valuation. Thankfully, initiatives led by cardiothoracic surgeons and anesthesiologists began to turn the tide.

On the cardiothoracic surgery side, initial initiatives were largely led by Dr. Nevin Katz, a surgeon with an interest in critical care [2,9,17,32]. In 2004, he launched the first international continuing medical education conference titled “Cardiothoracic Surgical Critical Care: A Cross-Disciplinary Specialty of Evolving Concepts and Therapeutics.” This was followed by the establishment of the Foundation for Advancement of Cardio-Thoracic Surgical Care (FACTS-Care) [32]. With his help, the American Board of Thoracic Surgery (ABTS) considered the creation of a certification in cardiothoracic critical care in 2008 but ultimately decided against it in part due to the deficiency of “extensive credibility in critical care education” [7,9]. Nonetheless, a sample curriculum and core competencies for CT-CCM were developed and published by Dr. Hisham Sherif [33]. Alongside, the American Board of Surgery (ABS) agreed to allow interested critical care fellowship trainees to spend 4–6 months in the CT-ICU during ABS critical care fellowship training, making these attractive pathways for cardiothoracic surgeons who plan to practice CT-CCM [7,33]. FACTS-Care has transferred the management of yearly meetings to The Society of Thoracic Surgeons (STS), and its president, Dr. Katz, joined a consulting organization with the goals of creating global, multidisciplinary critical care education [34]. Tangible results from the last 20 years of innovation include the continuation of the annual conference, now entitled “Perioperative and Critical Care Conference”, ongoing advanced CT-CCM fellowships at Johns Hopkins and Cleveland Clinic, and the approval of customizable surgical critical care fellowships by the ABS [35,36,37].

Anesthesiologists have been an integral part of cardiothoracic surgery from its inception and similarly have assumed leading intensivist roles in the CT-ICUs in step with their surgical colleagues [1,15,30,34,38]. In fact, nearly 70% of critical care anesthesiologists practice in CT-ICUs [10]. This outcome is born from dedicated training at the residency level. Anesthesiology residents are required to have a minimum of 4-months of critical care experience during training and a minimum of 2-months of cardiothoracic anesthesia experience; this is coupled with the need to exhibit proficiency in basic transthoracic and transesophageal echocardiography at the end of the residency [39,40]. As a comparison, neither internal medicine (IM) nor general surgery residencies have such stringent requirements, both in critical care exposure or towards focused learning in cardiothoracic care [41,42].

Anesthesiology-led fellowship training has also been adjusted to address future critical care needs [43]. The number of critical care anesthesiology fellowship positions and programs has increased significantly over the last decade, more than doubling the available training spots [44]. Supplementing this, multiple centers now offer residency positions automatically linked to the critical care fellowship acceptance [45,46]. Finally, emphasis on skills unique to CT-ICU has also paved the way for dual-fellowship training in cardiothoracic and critical care anesthesiology. Graduates with these credentials have expanded markedly, and dual-fellowship positions are now routinely offered, with hopes to integrate the training pathway in the upcoming years [47].

## 4. Why Is CT-CCM Specific Training Needed?

As the complex perioperative environment of cardiothoracic and vascular surgery evolves, so does the need for physicians specialized in managing these critically ill populations [14]. The wisdom and competence required to navigate the difficult perioperative ecosystem of a CT-ICU are laborious to develop and surpass the learning of any single skill or ability. Current CT-ICU practices are the pinnacle of medical science, integrating core critical care concepts with evolving devices, extra-corporeal support, and transplant considerations. Extra-corporeal life support (ECLS), durable mechanical circulatory support (DMCS), cardiac surgical unit advanced life support (CSU-ALS), point-of-care ultrasonography (POCUS), ethical challenges, and team management are just a few examples of complexities encountered in CT-ICUs. This section describes unique aspects of CT-CCM with specific examples that exemplify why specific, focused training is necessary.

### 4.1. ECLS

ECLS is primarily utilized and managed in CT-ICUs [18]. In effect, best trained CT-ICU intensivists must be ECLS specialists with an intimate familiarity in patient selection, cannulation strategies, management, and destination therapy selection. This unique intersection between critically ill patients and the utilization of extra-corporeal membrane oxygenation (ECMO) was fully realized through the efforts of cardiothoracic intensivists during the COVID-19 pandemic [48]. CT-ICU physicians triaged patients for ECMO candidacy, in some centers, cannulated them, then managed the ECMO circuit, and finally, assisted with patient selection for lung transplantation [45,48,49]. They also provided specific scientific expertise related to ECMO physiology. A particular example of CT-CCM competence benefiting patient care was the management of hypoxemia in patients with COVID-19 supported with ECMO. Many physicians followed absolute oxygen saturations and partial pressure of oxygen values while on ECMO instead of using oxygen carrying capacity, delivery, and consumption [50,51]. This may have resulted in the unnecessary treatment of hypoxia with heavy sedation, prone positioning, and beta-blocker therapy, even when oxygen delivery met metabolic demands [50,51]. Distinct knowledge possessed by well-trained CT-ICU intensivists allowed for the avoidance of these unnecessary maneuvers and their associated risks [52].

However, the COVID-19 disease plays only a small part in overall competence in ECMO support. Many other complexities exist. For example, CT-ICU physicians need to be versed in the components and various configurations of ECMO for both respiratory and circulatory failure [53,54]. An appreciation of the difference between cannulation arrangements, such as veno-arterial-venous (VAV) vs. veno-venous-arterial (VVA), is crucial; although seemingly similar in terminology, the indications and effects of each configuration are strikingly different [55]. Additionally, the optimal use of other life support devices, including mechanical ventilation and renal replacement therapies, requires an understanding of the interactions among them and how to optimize their use to ensure the best outcomes for the patient and avoid unnecessary risk. For example, the strict optimization of lung rest and the avoidance of ventilator-induced lung injury for patients with respiratory failure on ECMO is crucial for improving outcomes [56]. Moreover, drug sequestration in the circuit and oxygenator is a known issue which can alter the pharmacokinetics and pharmacodynamics of frequently used medications [57,58]. Knowledge of pharmacologic interactions of the ECMO circuit while tailoring medical therapeutics in line with best practices in critical care medicine is essential for clinicians caring for ECMO patients. Finally, while rare, the CT-ICU clinician must be able to respond rapidly to mechanical emergencies related to the circuit that are life-threatening, which include but are not limited to: circuit disruption, oxygenator failure, raceway rupture, cavitation, system or component alarm and failure, air embolism, and inadvertent decannulation and clots [59].

As ECLS services continue to expand, standardized education and training are required to provide clinical quality and competency control. Certification exams and workshops delivered by the Extracorporeal Life Support Organization (ELSO) are a good start in this process, but insufficient to truly train a competent practitioner. Extended, hands-on clinical training and mentorship in concert with a certification process are a priority.

### 4.2. CSU-ALS

The complexities of care in the CT-ICU are not just limited to mechanical devices and organ failures. Advanced cardiac life support (ACLS) after cardiac surgery differs from ACLS provided in any other setting. CSU-ALS is an altered ACLS protocol explicitly designed for the resuscitation of patients after cardiac surgery [60]. Some important differences include an initial deferral of chest compressions in favor for the fast defibrillation of malignant arrhythmias, reduction of epinephrine bolus doses, disconnection of pacer wires, cessation of infusions, prompt chest opening for manual cardiac massage and tamponade relief, and rapid application of ECLS, if necessary [60]. Multidisciplinary teams adapted to the new ACLS paradigm are required to certify, display appropriate skills and competence, and recertify every 2-years. With more data coming out relating to the benefits of CSU-ALS, training physicians with an intimate knowledge and interest in these procedures is paramount for patient care and the advancement of research in the perioperative resuscitation of cardiac surgery patients.

### 4.3. POCUS

The role of critical care ultrasonography in diagnosing and managing CTICU patients continues to expand [61]. With the recent improvement in the availability of surface probes and the portability of ultrasound machines, the indications for use in CT-CCM have extended beyond cardiac assessment. Focused clinical assessments of the lungs, diaphragm, airway, gallbladder, kidneys, blood vessels, and optic nerve sheaths have been increasingly utilized in critical care [61]. This advancement has now transformed bedside diagnostics. For example, ultrasound is now more sensitive for diagnosing a pneumothorax than a chest radiograph [62]. Yet another example, the size of the optic nerve sheath can be used for the evaluation of an intracranial pressure [63]. These skills are fundamental to patient care in current CT-ICUs.

### 4.4. Team and Leadership Skills

In the highly specialized ecosystem of CT-CCM, knowledge is crucial, but insufficient for the successful maturation of a competent CT-CCM intensivist. CT-CCM training must also tackle scientific literacy, ethical conduct, managerial competence, and interpersonal skills, as these are especially important for care excellence in this setting [14,49,64,65]. CT-CCM requires special scientific considerations and inquiry as the pathophysiology and mechanisms of injury often differ from patient populations in other ICUs. Consequently, CT-CCM is in dire need of physician-scientists who can answer the most pressing CT-ICU questions. Training physicians in an environment where CT-CCM distinction is appreciated is likely to produce intensivists interested in expanding knowledge unique to the field. Additionally, ensuring intimate familiarity with four ethical principles and their application in CT-ICU is paramount. CT-ICUs are especially ethically challenging due to high risk of complications and mortality [66]. Moreover, significant resource utilization, 30-day public mortality reporting, and the emotional involvement of the medical team can affect shared decision-making [67,68]. As a result, trainees need to learn how to navigate ethically difficult situations specific to CT-ICUs. Lastly, managerial skills are a necessity given the expansion of advanced practice practitioners and their strong presence in surgical teams; intensivists need to be trained with ample yet supportive supervision [69]. Additionally, juggling competing opinions from all of the stakeholders along with conflict management are crucial for patient safety, yet again highlighting the importance of executive competence [64,65].

In summary, developing wisdom and competency in CT-CCM require specific education and mentorship. CT-CCM is complex, challenging, and cognitively and emotionally taxing. Hence, subspecialty training is necessary to develop physicians who are competent intellectually and behaviorally to lead large, complex teams, provide the highest level of care, and advance CT-CCM as a medical science efficiently and effectively.

## 5. Goals of Standardized Training in CT-CCM

We propose an anesthesiology-led initiative creating standardized and supplementary training focused on the fundamentals of CT-CCM. The goals of this proposal are: (1) the improvement of patient outcomes by the standardization of clinical education, (2) the advancement of CT-CCM as a medical science and subspeciality of critical care, and (3) the support of physicians with on-the-job credentialing. Given the current fragmentation of critical care teaching, we recommend a curriculum open to individuals from any medical field who obtained critical care training from their specialty. The most sensible course of action may be the creation of a multidisciplinary, nationwide network of training programs responsible for the advancement and education of CT-CCM. We believe that anesthesiologists are best suited to lead such initiatives due to existing cardiothoracic and critical care expertise, as well as the largest intensivist representation in the CT-ICUs out of all specialties. With the guidance of educational experts, we would create a much-needed diverse partnership with all stakeholders based on mutual recognition of the importance, as opposed to previous hierarchical structures of the 20th century. The existence of two such programs assures us that further development and expansion would be met with interest [35,36].

1. Standardized Curriculum/Supplementary Training

A model CT-CCM curriculum would concentrate on the complexities of care not encountered in general critical care training with the goals of preparing physicians to provide consistent, safe, evidence-based, and cost-effective care [33]. In recognition of the complex care required in this patient population, training and experience would cover specialized medical knowledge, technical skills, and clinical acumen. Overall training would be distinct in focus from the independent completion of critical care and cardiothoracic anesthesiology fellowships. At the end of such training, a physician would display the competencies listed in Table 1.

Ostensibly, trainees would spend most of their time in the CT-ICU. In this setting, they would work with surgeons, specialist consultants, other medical professionals, and trainees. Since not all fellows will necessarily have gone through an anesthesiology or surgical residency, exposure to the cardiothoracic operating room could be included to provide foundational familiarity with the growing variety of procedures for other trainees. In addition, an anatomy lab course would provide a trainee with the basics of cardiothoracic procedural anatomy. Moreover, boot camps in ECLS, DMCS, POCUS, and CSU-ALS would be required at the initiation of the curriculum to lay down the fundamentals for the rest of the training. Furthermore, the introduction to a simulation center would help trainees become acclimated with this specific learning environment. Finally, the assessment of knowledge would be gaged with an entrance competency exam and exit competency exam.

2. Advancement of CT-CCM as a Science and Subspeciality of Critical Care Medicine

Scientific knowledge of CT-CCM is dispersed over many societies, journals, and specialties. The lack of a unified front and compartmentalization of information curtails systematic inquiry. The official training and recognition of CT-CCM would create a pool of physicians with shared scientific interests and goals, promoting advancement in the field. This topic will be further addressed in an editorial titled The Future of Cardiothoracic Surgical Critical Care Medicine as a Science: A Call to Action in this issue of Medicina.

3. Credentialing Support

The present lack of CT-CCM certification makes establishing appropriate credentials for the CT-ICU challenging. Many anesthesiologists spend several months of training in the CT-ICU environment during their residencies and critical care fellowships. Those wishing to work in the CT-ICU environment may deliberately pursue both critical care and cardiothoracic anesthesia fellowships. CT-CCM training is considered inherent to the education of CT surgeons. However, there is concern that hospital leadership may favor non-surgeons with non-specific, but recognizable critical care training [6]. As stated earlier, there are only a handful of specific CT-CCM programs [35,36]. Hospitals looking to staff CT-ICUs with appropriately trained providers may look for qualifications such as the National Board of Echocardiography certification in critical care ultrasound or perioperative transesophageal echocardiography and the completion of the ELSO Adult ECMO Practitioner Certification (E-AEC).

The lack of standardized qualifications presents an opportunity for the American Board of Anesthesiologists to partner with other medical boards and societies to establish minimal criteria for common competence in the area of CT-CCM. Such an effort must include critical care providers from various backgrounds, such as anesthesiology, surgery, pulmonary critical care, the emerging area of cardiac critical care, [86] and emergency medicine. An additional process would need to allow providers currently working in the CT-ICUs to become credentialed. Establishing clear competencies for CT-CCM would support the growth of this subspecialty, guide future training, and promote consistent, effective, and efficient care for cardiothoracic surgery patients.

## 6. Conclusions

CT-CCM is a crucial component of perioperative patient care and requires special training to develop expertise and competency. At this time, the training of CT-CCM intensivists is non-standardized and scattered between multiple specialties. Consequently, CT-CCM training needs strong leadership to improve current processes of its intensivist training. Anesthesiologists are uniquely suited to excel in this ecosystem because of their training background and skillset. Leading the educational process of CT-CCM intensivists is a natural extension of current presence in the field of cardiothoracic surgery and intensive care. The goals of this leadership are the creation of a specific training curriculum resulting in improved patient care, the expansion of CT-CCM as a medical science, and physician credentialing support. The expected growth of ideas in and outside of the intensive care units would solidify CT-CCM as an official subspeciality of CCM and a unique medical science, improve patient outcomes, and aid with physician credentialing, creating the future of distinction of cardiothoracic surgical intensive care.

## Figures and Tables

**Table 1:** Competency Based Training–Core Skills and Competencies Acquired During CT-CCM Training.

Competency	Considerations
Clinical Knowledge	Core critical care competencies are mandated and can follow traditional guidance from ABA or other professional boards. Additional guidance is required within the CT-CCM, emphasizing core topics with specific goals, and objectives. These include but are not limited to: Expertise in open, minimally invasive, and percutaneous cardiothoracic and vascular procedures, including patient presentation, selection, intraprocedural, and postoperative course Identification, evaluation, and management of postoperative complications CT-CCM specific pharmacology Evaluation and management of neurologic complications common to CT-CCM Identification and management of septic shock in CT-CCM patients Identification, evaluation, and management of cardiogenic shock Identification and management of vasoplegia Management of mechanical ventilation in ARDS Renal replacement therapy in CT-CCM specific population Transfusion medicine in CT-CCM specific population Lumbar drain indications and management
CSU-ALS	ACLS specific to the cardiothoracic population entails unique diagnostic, management, and procedural considerations [60,70]. Formal training and high-level competency testing are paramount.
Ultrasonography	Multiple pathways exist towards training in critical care echocardiography. Transesophageal echocardiography certifications are available and encouraged, but Certification in Critical Care Ultrasonography is required [71,72]. Additional expertise in POCUS including lung ultrasound and other detailed assessments is paramount (e.g., FAST windows, ultrasound-guided line placement and procedural guidance, ICP, and many more). Fluency in all the above is required to diagnose acute problems and titrate therapies in the CT-ICU.
Hemodynamic Monitoring	Advanced hemodynamic monitoring and support devices should be able to be placed and interpreted, including arterial lines, central venous lines, pulmonary artery catheters. IABP placement, removal, and management is similarly mandated.
Mechanical Support including ECMO	In-depth understanding, including patient candidacy, device selection, cannulation strategies, management, complications, troubleshooting, and weaning is required for both durable and temporary extra-corporeal support devices, including both VV- and VA-ECMO, VAD and the total artificial heart. The INTERMACS categorization system should be understood [69]. Fluency in pathways for patients who fail to wean must be achieved. Initiation and management of ECMO and other ECLS use for advanced respiratory and cardiac failure, including E-CPR, is necessary [73,74,75].
Palliative and End-of-Life Care	Palliative issues specific for postoperative patients and specific to CTICU patients, including patients on mechanical support, are necessary, and fluency is required to bridge the gap between patient and surgeon [76,77,78,79,80].
Organ Transplantation	Active participation in organ transplantation selection committees would offer trainees intimate understanding of the processes required for a successful organ placement. Appreciation of preoperative factors, including mental health, cognitive function, physical status, social support, and chronic disease burden would provide long-term perspective, and patient continuity often lacking in an ICU. Selection criteria for both heart and lung transplantation need to be understood intimately.
Post-ICU Care	Knowledge of post-intensive care unit syndrome and other important facets of the prolonged recovery process specific to CT-ICU population are necessary [81].
Leadership, Communication and Behavioral Skills	Excellence in CT-CCM requires broad involvement of interdisciplinary practitioners from varied backgrounds. Effective communication, teamwork, conflict management, advanced practice provider supervision, and leadership are mandated to successfully care for this complex population [82].
Research, Quality Improvement and Other Topics	Standardized curricula exist in critical care research, statistics, and quality improvement. Ongoing support should be available, and competency should be mandated in interpretation of active literature. Research in CT-CCM should be strongly encouraged. An in-depth understanding of medical systems, referral processes and inter-hospital issues, and finances of healthcare are necessary. Standardizing care to achieve pre-determined outcomes defined by professional societies, such as the Society of Thoracic Surgery (STS) or ELSO, is an important component of CT-CCM quality of care (QOI) [83]. Development of multidisciplinary protocols can decrease adverse outcomes, such as acute kidney injury, bleeding, and mortality [20]. Important ongoing areas of study include the impact of non-cardiovascular disease on this patient population, predicting outcomes, identifying optimal timing for interventions, and implementation of protocols to improve consistency of care [84]. CT-CCM trainees need to be actively involved in learning and practicing QOI skills.
Assessment of Skills	In-training examinations and simulation can be utilized to assess skill, maintain a high standard of excellence, and confirm capabilities. Early outcomes can dictate the time required for successful independence practice in the ICU [85]. Ongoing CME specific to the CTICU should be required and can be integrated into existing MOCA^®^ models.

ABA, American Board of Anesthesiology; ACLS, advanced cardiac life support; CALS, cardiac advanced life support; CME, continuing medical education; ECLS, extra-corporeal life support; ECPR, extra-corporeal cardiopulmonary resuscitation; ECMO, extra-corporeal membrane oxygenator; FAST, focused assessment with sonography for trauma; IABP, intraaortic balloon pump; INTERMACS, interagency registry for mechanically assisted circulatory support; MOCA, Maintenance of Certification in Anesthesia^®^; VAD, ventricular assist device.

## Data Availability

Not applicable.

## References

[1] Andrews M.C., Ivascu N.S., Pearl R.G. (2017). Cardiothoracic Critical Care: A New Specialty. ASA Monit..

[2] Katz N.M. (2015). Meeting the expanded challenges of the cardiothoracic intensive care unit. J. Thorac. Cardiovasc. Surg..

[3] Sherif H.M. (2016). Cardiothoracic surgical critical care surgeons: Many of the few. J. Thorac. Cardiovasc. Surg..

[4] Sherif H.M. (2016). Cardiothoracic surgical critical care certification: A future of distinction. J. Thorac. Cardiovasc. Surg..

[5] Stahl D.L., Ivascu N.S., Ben-Jacob T.K., Goff K. (2019). Beyond the Operating Room: Choosing a Career in Critical Care. ASA Monit..

[6] Andersen N.D. (2016). Certification in cardiothoracic surgical critical care: A distinction for some or for all?. J. Thorac. Cardiovasc. Surg..

[7] Calhoon J.H., Shemin R.J., Allen M.S., Baumgartner W.A. (2013). The American board of thoracic surgery: Update. Ann. Thorac. Surg..

[8] Sherif H.M. (2012). Cardiothoracic surgical critical care: Principles, goals and direction. Int. J. Surg..

[9] Katz N.M. (2013). It is time for certification in cardiothoracic critical care. J. Thorac. Cardiovasc. Surg..

[10] Shaefi S., Pannu A., Mueller A.L., Flynn B., Evans A., Jabaley C.S., Mladinov D., Wall M., Siddiqui S., Douin D.J. (2022). Nationwide Clinical Practice Patterns of Anesthesiology Critical Care Physicians—A Survey to Members of the Society of Critical Care Anesthesiologists. Anesth. Analg..

[11] Verdiner R.E., Choukalas C.G., Siddiqui S., Stahl D.L., Galvagno S.M., Jabaley C.S., Bartz R.R., Lane-Fall M., Goff K.L., Sreedharan R. (2020). Narrative Review Article: Coronavirus Disease–Activated Emergency Scaling of Anesthesiology Responsibilities Intensive Care Unit. Anesth. Analg..

[12] American Society of Anesthesiologists (2020). APSF/ASA Guidance on Purposing Anesthesia Machines as ICU Ventilators.

[13] Arora L., Pannu A., Bose S. (2021). ECMO in the COVID-19 Era: Looking Beyond the Guidelines. ASA Monit..

[14] Bartels K., Dieleman S.J. (2019). Cardiothoracic Anesthesia and Critical Care: An Ever-Changing (and Evolving) Field. Anesthesiol. Clin..

[15] Weiss S.J. (2004). Pro: Cardiothoracic anesthesiologists should run postcardiac surgical intensive care units. J. Cardiothorac. Vasc. Anesth..

[16] Arora R.C., Hirose H., Engelman D., Rabin J., Firstenberg M., Moosdorf R.G., Geller C.M., Hiebert B. (2020). Survey of contemporary cardiac surgery intensive care unit models in the United States. Ann. Thorac. Surg..

[17] Katz N.M. (2011). The evolution of cardiothoracic critical care. J. Thorac. Cardiovasc. Surg..

[18] Owyang C.G., Donnat C., Brodie D., Gershengorn H.B., Hua M., Qadir N., Tonna J.E. (2022). Similarities in extracorporeal membrane oxygenation management across intensive care unit types in the United States: An analysis of the Extracorporeal Life Support Organization Registry. Artif. Organs.

[19] Kumar K., Zarychanski R., Bell D.D., Manji R., Zivot J., Menkis A.H., Arora R. (2009). Impact of 24-hour in-house intensivists on a dedicated cardiac surgery intensive care unit. Ann. Thorac. Surg..

[20] Stamou S.C., Camp S.L., Stiegel R.M., Reames M.K., Skipper E., Watts L.T., Nussbaum M., Robicsek F., Lobdell K.W. (2008). Quality improvement program decreases mortality after cardiac surgery. J. Thorac. Cardiovasc. Surg..

[21] Kumar K., Singal R., Manji R.A., Zarychanski R., Bell D.D., Freed D.H., Arora R.C. (2014). The benefits of 24/7 in-house intensivist coverage for prolonged-stay cardiac surgery patients. J. Thorac. Cardiovasc. Surg..

[22] Benoit M.A., Bagshaw S.M., Norris C.M., Zibdawi M., Chin W.D., Ross D.B., van Diepen S. (2017). Postoperative complications and outcomes associated with a transition to 24/7 intensivist management of cardiac surgery patients. Crit. Care Med..

[23] Huard P., Kalavrouziotis D., Lipes J., Simon M., Tardif M.A., Blackburn S., Langevin S., Sia Y.T., Mohammadi S. (2020). Does the full-time presence of an intensivist lead to better outcomes in the cardiac surgical intensive care unit?. J. Thorac. Cardiovasc. Surg..

[24] Lee L.S., Clark A.J., Namburi N., Naum C.C., Timsina L., Corvera J.S., Beckman D.J., Everett J.E., Hess P.J. (2020). The presence of a dedicated cardiac surgical intensive care service impacts clinical outcomes in adult cardiac surgery patients. J. Card. Surg..

[25] Kim D.J., Sohn B., Kim H., Chang H.W., Lee J.H., Kim J.S., Lim C., Park K.-H. (2020). The Impact of an Attending Intensivist on the Clinical Outcomes of Patients Admitted to the Cardiac Surgical Intensive Care Unit after Coronary Artery Bypass Grafting. Korean J. Thorac. Cardiovasc. Surg..

[26] Wu H.Y., Chao H.-S., Shih H.-C., Shih C.-C., Chang S.-C. (2018). The impact of open to collaborative care model in cardiovascular surgical unit. J. Chin. Med. Assoc..

[27] Ramsay J. (2004). Con: Cardiothoracic anesthesiologists should not run postcardiac surgical intensive care units. J. Cardiothorac. Vasc. Anesth..

[28] Whitson B.A., D’Cunha J. (2011). The thoracic surgical intensivist: The best critical care doctor for our thoracic surgical patients. Seminars in Thoracic and Cardiovascular Surgery.

[29] Whitman G.J.R. (2011). Cardiothoracic surgeon management of postoperative cardiac critical care. Arch. Surg..

[30] Whelan S.E. (2007). The Sisters’ Story: Saint Marys Hospital-Mayo Clinic, 1939 to 1980.

[31] Hilberman M. (1975). The evolution of intensive care units. Crit. Care Med..

[32] Katz N.M. (2007). The emerging specialty of cardiothoracic surgical critical care: The leadership role of cardiothoracic surgeons on the multidisciplinary team. J. Thorac. Cardiovasc. Surg..

[33] Sherif H.M. (2012). Developing a curriculum for cardiothoracic surgical critical care: Impetus and goals. J. Thorac. Cardiovasc. Surg..

[34] About U.S. (2022). The Foundation for the Advancement of Cardio-Thoracic Surgical Care. https://www.facts-care.org/cardiac-care/.

[35] (2022). Cardiothoracic Intensive Care Fellowship. https://my.clevelandclinic.org/departments/anesthesiology/medical-professionals/fellowships/cardiothoracic-intensive-care#overview-tab.

[36] (2022). Cardiac Surgery Critical Care Fellowship. https://www.hopkinsmedicine.org/fellowships/programs/heart-vascular-institute/cardiac-critical-care-fellowship.

[37] (2022). Surgical Critical Care Tracks. https://uwsurgery.org/divisions/trauma-and-burn/education-2/surgical-critical-care-tracks/.

[38] Mehta Y. (2015). Is cardiac anaesthesiologist the best person to look after cardiac critical care?. Ann. Card. Anaesth..

[39] (2022). Anesthesiology Program Requirements and FAQs. https://www.acgme.org/globalassets/pfassets/programrequirements/040_anesthesiology_2022.pdf.

[40] (2022). OSCE Content Outline. https://www.theaba.org/pdfs/OSCE_Content_Outline.pdf.

[41] (2022). General Surgery Program Requirements and FAQs. https://www.acgme.org/globalassets/pfassets/programrequirements/440_generalsurgery_2022.pdf.

[42] (2022). Internal Medicine Program Requirements and FAQs. https://www.acgme.org/globalassets/pfassets/programrequirements/140_internalmedicine_2022v4.pdf.

[43] Irwin R.S., Marcus L., Lever A. (2004). special reports. Chest.

[44] Khanna A.K., Siddiqui S., Kaufman M., Grecu L. (2019). Anesthesiologist Intensivists: Adding Value to a Hospital System. ASA Monit..

[45] Nalley C. (2021). Massachusetts General Hospital: A Long History of Innovation and Impact. ASA Monit..

[46] (2022). Critical Care Scholars. https://anesthesia.ucsf.edu/critical-care-scholars.

[47] Bhatia M. (2018). Fellowship Spotlight: Cardiothoracic and Critical Care Anesthesia. https://www.asahq.org/education-and-career/asa-resident-component/resident-newsletters/fellowship-spotlight---cardiothoracic-and-critical-care-anesthesia.

[48] Wolfgang K. (2022). Anesthesiologists Drive Expanded Use of ECMO. ASA Monit..

[49] Shelton K.T., Wiener-Kronish J.P. (2020). Evolving role of anesthesiology intensivists in cardiothoracic critical care. Anesthesiology.

[50] Kopanczyk R., Bhatt A., Kumar N., Henson C.P. (2022). Persistent hypoxemia in COVID-19 patients on ECMO: Keep your eyes on the prize. J. Cardiothorac. Vasc. Anesth..

[51] Ortoleva J.P., Chweich H. (2022). Normalizing the Abnormal: Hypoxemia in Venovenous ECMO. J. Cardiothorac. Vasc. Anesth..

[52] Gurnani P.K., Michalak L.A., Tabachnick D., Kotwas M., Tatooles A.J. (2022). Outcomes of Extubated COVID and Non-COVID Patients Receiving Awake Venovenous Extracorporeal Membrane Oxygenation. ASAIO J..

[53] Le Gall A., Follin A., Cholley B., Mantz J., Aissaoui N., Pirracchio R. (2018). Veno-arterial-ECMO in the intensive care unit: From technical aspects to clinical practice. Anaesth. Crit. Care Pain Med..

[54] DeFilippis E.M., Clerkin K., Truby L.K., Francke M., Fried J., Masoumi A., Garan A.R., Farr M.A., Takayama H., Takeda K. (2021). ECMO as a bridge to left ventricular assist device or heart transplantation. Heart Fail..

[55] Napp L.C., Kühn C., Hoeper M., Vogel-Claussen J., Haverich A., Schäfer A., Bauersachs J. (2016). Cannulation strategies for percutaneous extracorporeal membrane oxygenation in adults. Clin. Res. Cardiol..

[56] Marhong J.D., Telesnicki T., Munshi L., Del Sorbo L., Detsky M., Fan E. (2014). Mechanical ventilation during extracorporeal membrane oxygenation. An international survey. Ann. Am. Thorac. Soc..

[57] Shekar K., A Roberts J.A., I Mcdonald C., Ghassabian S., Anstey C., Wallis S.C., Mullany D.V., Fung Y.L., Fraser J.F. (2015). Protein-bound drugs are prone to sequestration in the extracorporeal membrane oxygenation circuit: Results from an ex vivo study. Crit. Care.

[58] Shekar K., Roberts J.A., I Mcdonald C., Fisquet S., Barnett A.G., Mullany D.V., Ghassabian S., Wallis S.C., Fung Y.L., Smith M.T. (2012). Sequestration of drugs in the circuit may lead to therapeutic failure during extracorporeal membrane oxygenation. Crit. Care.

[59] Burrows P.E., Mason K.P. (2004). Percutaneous treatment of low flow vascular malformations. J. Vasc. Interv. Radiol..

[60] Dunning J., Levine A., Ley J., Strang T., Lizotte D.E., Lamarche Y., Bartley T., Zellinger M., Katz N., Arora R.C. (2017). The society of thoracic surgeons expert consensus for the resuscitation of patients who arrest after cardiac surgery. Ann. Thorac. Surg..

[61] Kalagara H., Coker B., Gerstein N.S., Kukreja P., Deriy L., Pierce A., Townsley M.M. (2021). Point-of-care ultrasound (POCUS) for the cardiothoracic anesthesiologist. J. Cardiothorac. Vasc. Anesth..

[62] Ingelfinger J.R., José L., Díaz-Gómez M.D., Paul H., Mayo M.D., Seth J., Koenig M.D. (2021). Point-of-Care Ultrasonography. N. Engl. J. Med..

[63] Rasulo F.A., Bertuetti R. (2019). Transcranial Doppler and optic nerve sonography. J. Cardiothorac. Vasc. Anesth..

[64] Merry A.F., Weller J., Mitchell S.J. (2014). Teamwork, communication, formula-one racing and the outcomes of cardiac surgery. J. Extra-Corpor. Technol..

[65] Kayser J.B., Kaplan L.J. (2020). Conflict management in the ICU. Crit. Care Med..

[66] Kim J.M., Godfrey S., O’Neill D., Sinha S.S., Kochar A., Kapur N.K., Katz J.N., Warraich H.J. (2022). Integrating palliative care into the modern cardiac intensive care unit: A review. Eur. Heart J. Acute Cardiovasc. Care.

[67] Turnbull A.E., Sahetya S.K., Biddison E.L.D., Hartog C.S., Rubenfeld G.D., Benoit D.D., Guidet B., Gerritsen R.T., Tonelli M.R., Curtis J.R. (2018). Competing and conflicting interests in the care of critically ill patients. Intensive Care Med..

[68] Schwarze M.L., Brasel K.J., Mosenthal A.C. (2014). Beyond 30-day mortality: Aligning surgical quality with outcomes that patients value. JAMA Surg..

[69] Rincon T.A., Bakshi V., Beninati W., Carpenter D., Cucchi E., Davis T.M., Dreher J., Hiddleson C., Johansson M.K., Katz A.W. (2020). Describing advanced practice provider roles within critical care teams with tele-ICUs: Exemplars from seven US health systems. Nurs. Outlook.

[70] Dunning J., Nandi J., Ariffin S., Jerstice J., Danitsch D., Levine A. (2006). The Cardiac Surgery Advanced Life Support Course (CALS): Delivering significant improvements in emergency cardiothoracic care. Ann. Thorac. Surg..

[71] Panebianco N.L., Mayo P.H., Arntfield R.T., Brown S.M., Diaz-Gomez J., Hernandez A., Koenig S.J., Noble V., Sekiguchi H., Subhiyah R.G. (2021). Assessing Competence in Critical Care Echocardiography: Development and Initial Results of an Examination and Certification Processes. Crit. Care Med..

[72] National Board of Echocardiography (2021). Handbooks for NBE Certification Programs. https://www.echoboards.org/EchoBoards/Certifications/Certification/Handbooks_for_All_Certification_Programs/EchoBoards/Certifications/Handbooks_for_NBE_Certification_Programs.aspx.

[73] Combes A., Peek G.J., Hajage D., Hardy P., Abrams D., Schmidt M., Dechartres A., Elbourne D. (2020). ECMO for severe ARDS: Systematic review and individual patient data meta-analysis. Intensive Care Med..

[74] Dennis M., Lal S., Forrest P., Nichol A., Lamhaut L., Totaro R.J., Burns B., Sandroni C. (2020). In-depth extracorporeal cardiopulmonary resuscitation in adult out-of-hospital cardiac arrest. J. Am. Heart Assoc..

[75] Richardson A.S.C., Tonna J.E., Nanjayya V., Nixon P., Abrams D.C., Raman L., Bernard S., Finney S.J., Grunau B., Youngquist S.T. (2021). Extracorporeal cardiopulmonary resuscitation in adults. Interim guideline consensus statement from the extracorporeal life support organization. ASAIO J..

[76] Yefimova M., Aslakson R.A., Yang L., Garcia A., Boothroyd D., Gale R.C., Giannitrapani K., Morris A.M., Johanning J.M., Shreve S. (2020). Palliative care and end-of-life outcomes following high-risk surgery. JAMA Surg..

[77] Leeds H., Smith D. (2020). Palliative Care Involvement in Patients with Operative Mortality After Cardiac Surgery. Heart Surg. Forum.

[78] Nakagawa S., Hua M., Takayama H. (2018). Who is not comfortable with the term “palliative care”-patient, family, or surgeon?. J. Thorac. Cardiovasc. Surg..

[79] Katz N.M. (2018). The term “supportive care” is preferable to “palliative care” for consults in the cardiothoracic intensive care unit. J. Thorac. Cardiovasc. Surg..

[80] Smith A., Morgan C., Ledot S., Doyle J., Xu T., Shedden L., Passariello M., Patel B., Doyle A.M., Price S. (2021). Veno-venous extracorporeal membrane oxygenation for the acute respiratory distress syndrome: A bridge too far?. Acta Cardiol..

[81] Rousseau A.-F., Prescott H.C., Brett S.J., Weiss B., Azoulay E., Creteur J., Latronico N., Hough C.L., Weber-Carstens S., Vincent J.-L. (2021). Long-term outcomes after critical illness: Recent insights. Crit. Care.

[82] Kennedy-Metz L.R., Barbeito A., Dias R.D., Zenati M.A. (2022). Importance of high-performing teams in the cardiovascular intensive care unit. J. Thorac. Cardiovasc. Surg..

[83] Stephens R.S., Whitman G.J. (2015). Postoperative critical care of the adult cardiac surgical patient: Part II: Procedure-specific considerations, management of complications, and quality improvement. Crit. Care Med..

[84] Gilliland S., Tran T., Alber S., Krause M., Weitzel N. (2021). Year in Review 2020: Noteworthy Literature in Cardiothoracic Critical Care. Seminars in Cardiothoracic and Vascular Anesthesia.

[85] Castellanos-Ortega Á., Broch M.J., Palacios-Castañeda D., Gómez-Tello V., Valdivia M., Vicent C., Madrid I., Martinez N., Párraga M.J., Sancho E. (2022). Competency assessment of residents of Intensive Care Medicine through a simulation-based objective structured clinical evaluation (OSCE). A multicenter observational study. Med. Intensiva.

[86] Morrow D.A. (2017). Trends in cardiac critical care: Reshaping the cardiac intensive care unit. Circ. Cardiovasc. Qual. Outcomes.

